# Survival outcomes after surgery for type A aortic dissection: a contemporary Dutch nationwide registry study

**DOI:** 10.1093/icvts/ivaf009

**Published:** 2025-02-06

**Authors:** Patrick T Timmermans, Bart J J Velders, Rolf H H Groenwold, Roemer J Vos, Maaike M Roefs, Jerry Braun, Robert J M Klautz, Gianclaudio Mecozzi, Jesper Hjortnaes, Cardiothoracic Surgery Registration Committee and the Aortic Surgery Work Group of the Netherlands Heart Registration, Maaike Roefs, Gianclaudio Mecozzi, Jesper Hjortnaes

**Affiliations:** Department of Cardiothoracic Surgery, Leiden University Medical Center, Leiden, Netherlands; Department of Cardiothoracic Surgery, Leiden University Medical Center, Leiden, Netherlands; Department of Clinical Epidemiology, Leiden University Medical Center, Leiden, Netherlands; Department of Clinical Epidemiology, Leiden University Medical Center, Leiden, Netherlands; Department of Biomedical Data Science, Leiden University Medical Center, Leiden, Netherlands; Department of Cardiothoracic Surgery, Leiden University Medical Center, Leiden, Netherlands; Netherlands Heart Registration, Utrecht, Netherlands; Department of Cardiothoracic Surgery, Leiden University Medical Center, Leiden, Netherlands; Department of Cardiothoracic Surgery, Leiden University Medical Center, Leiden, Netherlands; Department of Cardiothoracic Surgery, University Medical Center Groningen, Groningen, Netherlands; Department of Cardiothoracic Surgery, Leiden University Medical Center, Leiden, Netherlands

**Keywords:** type A aortic dissection, nationwide registry, the Netherlands, cause of death

## Abstract

**OBJECTIVES:**

To describe the nationwide survival outcomes after surgery for type A aortic dissection.

**METHODS:**

All patients who underwent surgery for type A aortic dissection in the Netherlands between 2018 and 2021 were identified in the Netherlands Heart Registration (NHR) database. The NHR data were linked to lifelong survival data from Statistics Netherlands (98% match). Time trends for in-hospital and mid-term mortality were described, and age- and sex-adjusted regression analysis was performed. The cause and location of death were reported and stratified according to survival time intervals.

**RESULTS:**

The study population consisted of 1317 patients with a mean age of 63.1 years (11.8). The number of surgeries increased from 284 in 2018 to 375 in 2021. The surgery included the ascending aorta in 99%, aortic arch in 73%, aortic root in 32.5% and the descending aorta in 5% of cases. In-hospital mortality decreased from 20.4% in 2018 to 13.9% (95% CI: [10.4%, 17.4%]) in 2021. A total of 318 deaths were recorded, and the majority (70%) of patients died from the consequences of their dissection. However, among 365-day survivors, only 14% died related to their dissection, while 37% of deaths were related to cardiovascular disease and 17% to cancer. The majority (84%) of patients died in the hospital, but deaths after 365 days occurred most frequently (37%) at home.

**CONCLUSIONS:**

Over the recent years, the number of surgeries for type A dissections in the Netherlands has increased, and in-hospital mortality has decreased. For patients surviving 1 year after surgery, the main cause of death was not dissection, but other causes such as cardiovascular disease or cancer.

## Graphical abstract

**Figure:**
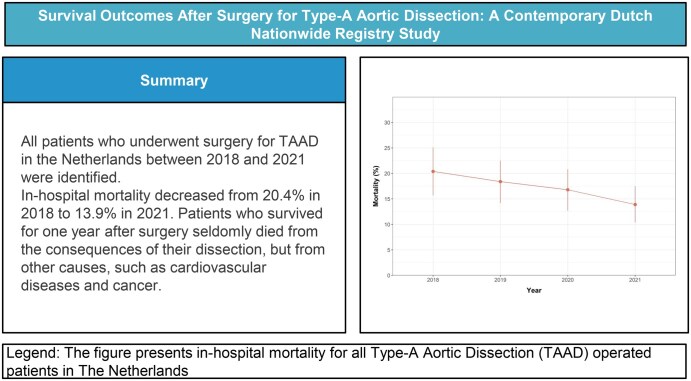

## INTRODUCTION

Acute type A aortic dissection (TAAD) is a medical emergency that requires urgent surgery [1–3]. Despite extensive research over the past decades, the in-hospital and early mortality rates for TAAD remain high [4–8]. Registries, such as the International Registry of Acute Aortic Dissection (IRAD) [6, 7, 9, 10], Nordic Consortium for Acute Type A Aortic Dissection (NORCAAD) [11, 12] and population-based cohorts such as the Oxford Vascular Study [13], Danish nationwide data [5, 14] and German Registry for Acute Aortic Dissection Type A (GERAADA) [15–17] provide important data on the incidence and outcomes of TAAD. These studies have analysed risk factors, surgical complications and mortality/reintervention rates; however, detailed information on the cause and location of death of TAAD is lacking. We use nationwide registry data to describe contemporary survival outcomes, including the cause and location of death, of all patients who underwent surgery for TAAD in the Netherlands between 2018 and 2021. These insights are valuable for both patients and for clinicians who provide treatment and follow-up.

## MATERIALS AND METHODS

### Ethics statement

This is an observational population-based cohort study of prospectively collected data registered within the Netherlands Heart Registration (NHR) and of data from Statistics Netherlands (‘Centraal Bureau voor de Statistiek (CBS)’ in Dutch). The study was approved by the institutional review board MEC-U (W19.270) and conducted in agreement with the principles of the Declaration of Helsinki. A waiver for informed consent was obtained. The data underlying this article were provided by the NHR with permission from all participating hospitals and by Statistics Netherlands; data are available upon reasonable request to the corresponding author and representatives of the data sources.

### Study data

The NHR is a nationwide, physician-driven and patient-focused quality registry that contains procedural and outcome data of all invasive cardiac interventional, electrophysiological and cardiothoracic surgical procedures from all Dutch hospitals [18, 19]. The aim of the NHR is to contribute to the maintenance and improvement of the quality and transparency of care for cardiac patients. For each intervention, a limited set of patient characteristics, procedural data and outcome data is collected by the hospitals and submitted to the NHR. Patients who underwent surgery for TAAD between 1 January 2018 and 31 December 2021 were selected. Reinterventions on the aorta were excluded. CBS contains, among other data outside the scope of this study, survival data of all inhabitants of the Netherlands. The NHR data were linked to data of Statistics Netherlands based on postal code and date of birth. From the NHR data, patient and procedural characteristics were obtained, while data from CBS were used to retrieve lifelong survival information including the date, cause and location of death. The causes of death were identified based on International Classification of Disease—10 (ICD-10) codes.

### Statistical analysis

All statistical analyses were performed using the R software (R Foundation for Statistical Computing, Vienna, Austria, www.r-project.org). Numerical variables were reported as mean (± standard deviation) or median (interquartile range) according to their distribution, and categorical variables as counts (percentages). Per the privacy regulations of Statistics Netherlands, cells were merged if they contained counts fewer than 5 to prevent possible identification of individuals. Time trends for in-hospital and mid-term mortality were graphically illustrated. Follow-up started on the day of surgery for TAAD. Furthermore, the age- and sex-adjusted associations between the mortality outcomes and the intervention years were quantified using binary logistic and Cox proportional hazards regression (R package *survival*). A graphical overview of the causes of death was provided for the entire study population, as well as for 30-day and 365-day survivors (R package mstate).

## RESULTS

In total, 1357 TAAD operations, performed between 1 January 2018 and 31 December 2021, were identified in the NHR database; 1325 (98%) of these could be matched to the CBS follow-up data based on postal code and date of birth. Of these 1325 operations, 8 (0.6%) were reoperations, leaving 1317 unique patients who underwent primary surgery for TAAD in The Netherlands. The number of TAAD operations increased over the 4-year study period: 284 in 2018, 332 in 2019, 328 in 2020 and 373 in 2021.

The average age at surgery was 63 ± 12 years, 59% was male, and the median EuroSCORE II was 10.1 [5.5, 19.7] (Table 1). Mean LVEF was 53%, and diabetes mellitus and chronic lung disease were reported in 4.2% and 6.8%, respectively. Sixty-four patients (7%) had connective tissue disease and 69 (5%) had a history of previous cardiac surgery; 392 patients (30%) were in a critical medical condition when they arrived in the operation room as defined by the need for cardiopulmonary resuscitation, intubation, inotropic support, or preoperative renal failure with urinary production <10 ml/h. Preoperative aspirin and direct oral anticoagulant were used by 12.5% and 8.4%, respectively.

**Table 1:** Baseline characteristics of 1317 patients who underwent surgery for acute type A aortic dissection in the Netherlands between 2018 and 2021

Characteristics	Overall	Missing data
Age (years)	63.1 (11.8)	0 (0%)
Male sex	770 (59%)	0 (0%)
BMI (kg/m^2^)	26.5 (4.7)	75 (6%)
BSA (m^2^)	2 (0.2)	75 (6%)
Intervention year		0 (0%)
2018	284 (22%)	
2019	332 (25%)	
2020	328 (25%)	
2021	373 (28.3%)	
EuroSCORE II	10.1 [5.5, 19.7]	106 (8%)
LVEF (%)	53.6 (6.4)	47 (4%)
Creatinine (μmol/l)	98 (47.1)	54 (4%)
Diabetes mellitus	54 (4%)	25 (2%)
Atrial fibrillation	104 (9%)	178 (14%)
Connective tissue disease	64 (7%)	367 (28%)
Chronic lung disease	89 (7%)	6 (1%)
Previous CVA	62 (5%)	17 (1%)
Previous ECVD	230 (18%)	6 (0.5%)
Previous cardiac surgery	69 (5%)	2 (0.2%)
Critical state	392 (30%)	3 (0.2%)
Urgency setting		0 (0%)
Elective	15 (1%)	
Urgent	110 (9%)	
Emergency	1016 (77%)	
Salvage	176 (13%)	
Preoperative aspirin use	128 (8%)	293 (22%)
Preoperative DOAC use	34 (4%)	350 (27%)

Data are presented as *n* (%); mean (±SD); median [interquartile range].

BMI: body mass index; BSA: body surface area; CVA: cerebrovascular accident; ECVD: extracardiac vascular disease; DOAC: direct oral anticoagulant; LVEF: left ventricular ejection fraction.

Regarding the procedural details (Table 2), the average cross-clamp time was 141 min, and 88% of the patients underwent circulatory arrest. Concomitant surgery on the aortic valve was performed in 39%. The valve was repaired in 11% and replaced in 28% (5% stentless bioprosthesis, 13% stented bioprosthesis and 10% mechanical prosthesis). Concomitant root surgery was performed in 32.5%, using either a Bentall, David or (partial) Yacoub procedure. Complete arch replacement (three vessels) was performed in 128 (10%) and zone 2 (2 vessels) arch replacement in 155 (12%). Six hundred thirty-five (49.6%) underwent hemi arch replacement and 317 (24%) underwent no arch surgery. In 61 (5%) patients, surgery was performed on the descending aorta, for example by using an (frozen) elephant trunk.

**Table 2:** Procedural details of 1317 patients who underwent surgery for acute type A aortic dissection in the Netherlands between 2018 and 2021

Characteristics	Overall	Missing data
ECC	1303 (99%)	4 (0.3%)
ECC time (min)	257.4 (101.5)	105 (8%)
Cross clamp time (min)	141.4 (69.4)	100 (8%)
Circulatory arrest	1148 (88.3%)	2 (0.2%)
Circulatory arrest time	43.6 (28.6)	159 (12%)
Concomitant CABG TAAD surgery extend	107 (8%)	0 (0%)
AV repair or replacement	520 (39%)	0 (0%)
Root	428 (32.5%)	45 (4%)
Aortic arch	961 (73%)	0 (0%)
Descending aorta	68 (5%)	1 (0.1%)
Minimum central temperature	23.4 (4)	192 (15%)
Number of arch vessels repaired		38 (3%)
0 vessels	953 (75%)	
1 vessel	42 (3%)	
2 vessels	155 (12%)	
3 vessels	128 (10%)	

Data are presented as *n* (%); mean (±SD). Times are reported in min.

AV: aortic valve; CABG: coronary artery bypass graft; ECC: extra corporeal circulation.

**Figure 1:**
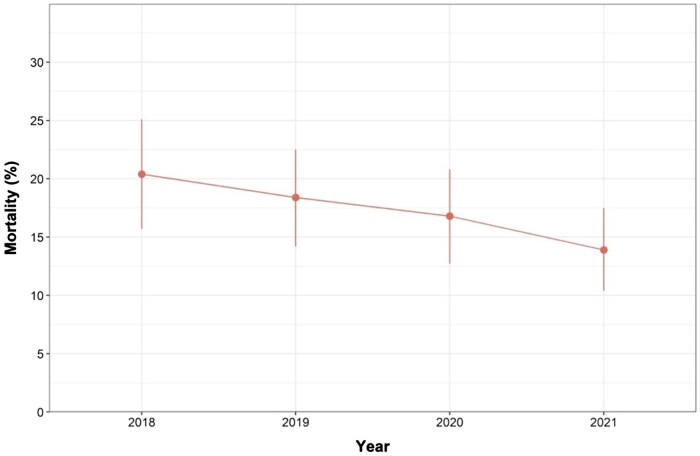
Yearly in-hospital mortality after surgery for acute type A aortic dissection in the Netherlands between 2018 and 2021. The dots represent the percentage of in-hospital mortality per intervention year, and the vertical bars represent the corresponding 95% confidence intervals.

**Figure 2:**
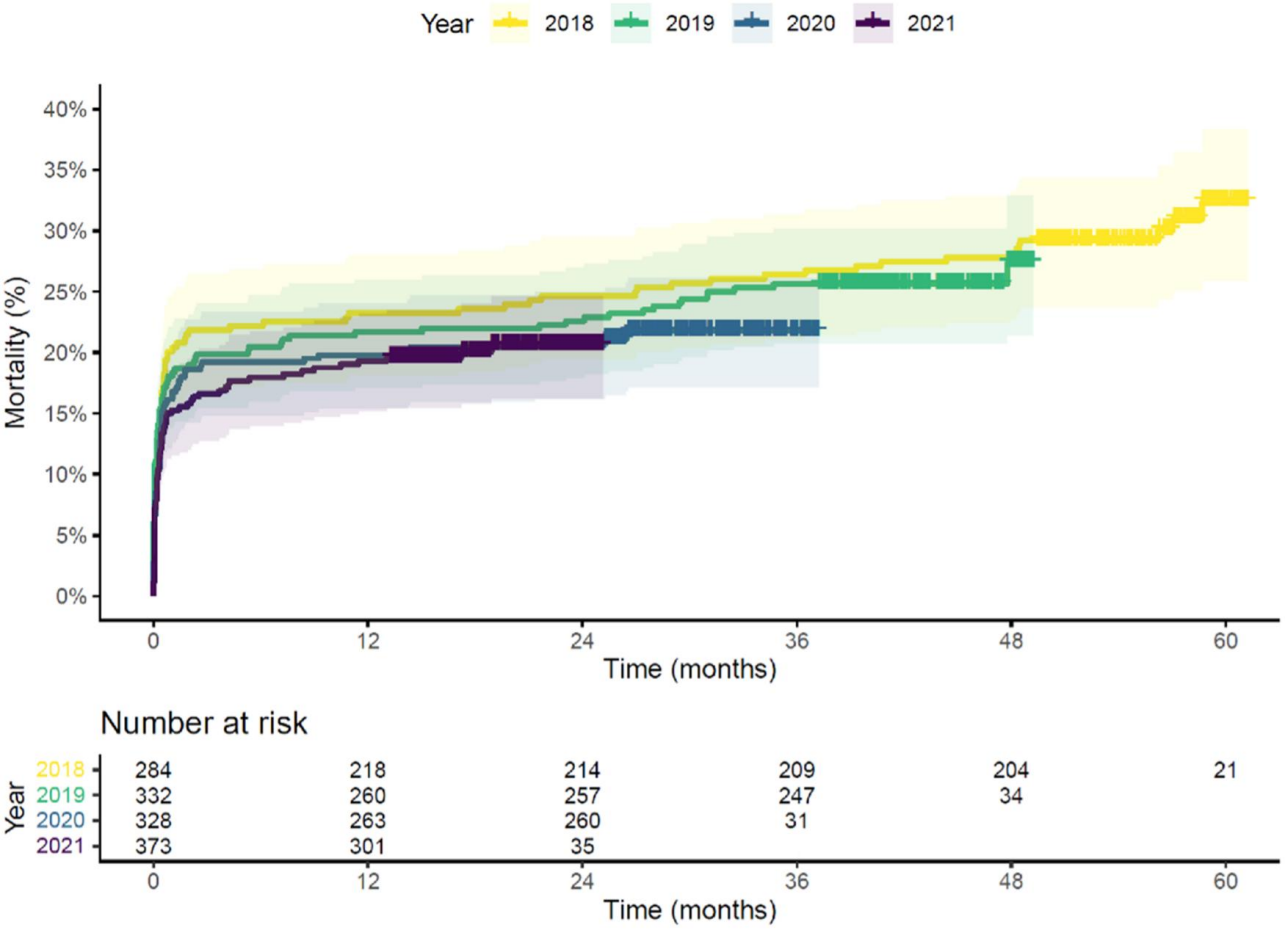
Kaplan–Meier survival curve per intervention year for patients who underwent surgery for acute type A aortic dissection in the Netherlands between 2018 and 2021. The coloured lines represent the cumulative incidences of mortality throughout 5-year follow-up, and the shaded areas represent the corresponding 95% confidence intervals. Censoring is indicated with the ‘+’ sign. Follow-up time started on the day of surgery.

The in-hospital mortality risk decreased over the 4-year study period (Fig. 1): 20.4% (95% confidence interval [CI] 13.8–25.1%) in 2018, 18.4% (95% CI 14.2–22.5) in 2019, 16.8% (95% CI 12.7–20.8%) in 2020 and 13.9% (95% CI 10.4–17.5%) in 2021. The age and sex-adjusted odds ratios for in-hospital mortality, with 2018 as reference, were 0.87 (95% CI 0.58–1.30) for 2019, 0.78 (95% CI 0.52–1.18) for 2020 and 0.63 (95% CI 0.41–0.95) for 2021 (Supplementary Material, Table S1). The mortality risk throughout the first 5 years after the index surgery is presented per intervention year in Fig. 2. The corresponding age- and sex-adjusted hazard ratios were 0.94 (95% CI 0.69–1.27) for 2019, 0.82 (95% CI 0.59–1.13) for 2020 and 0.82 (95% CI 0.59–1.12) for 2021 (Supplementary Material, Table S2).

**Figure 3:**
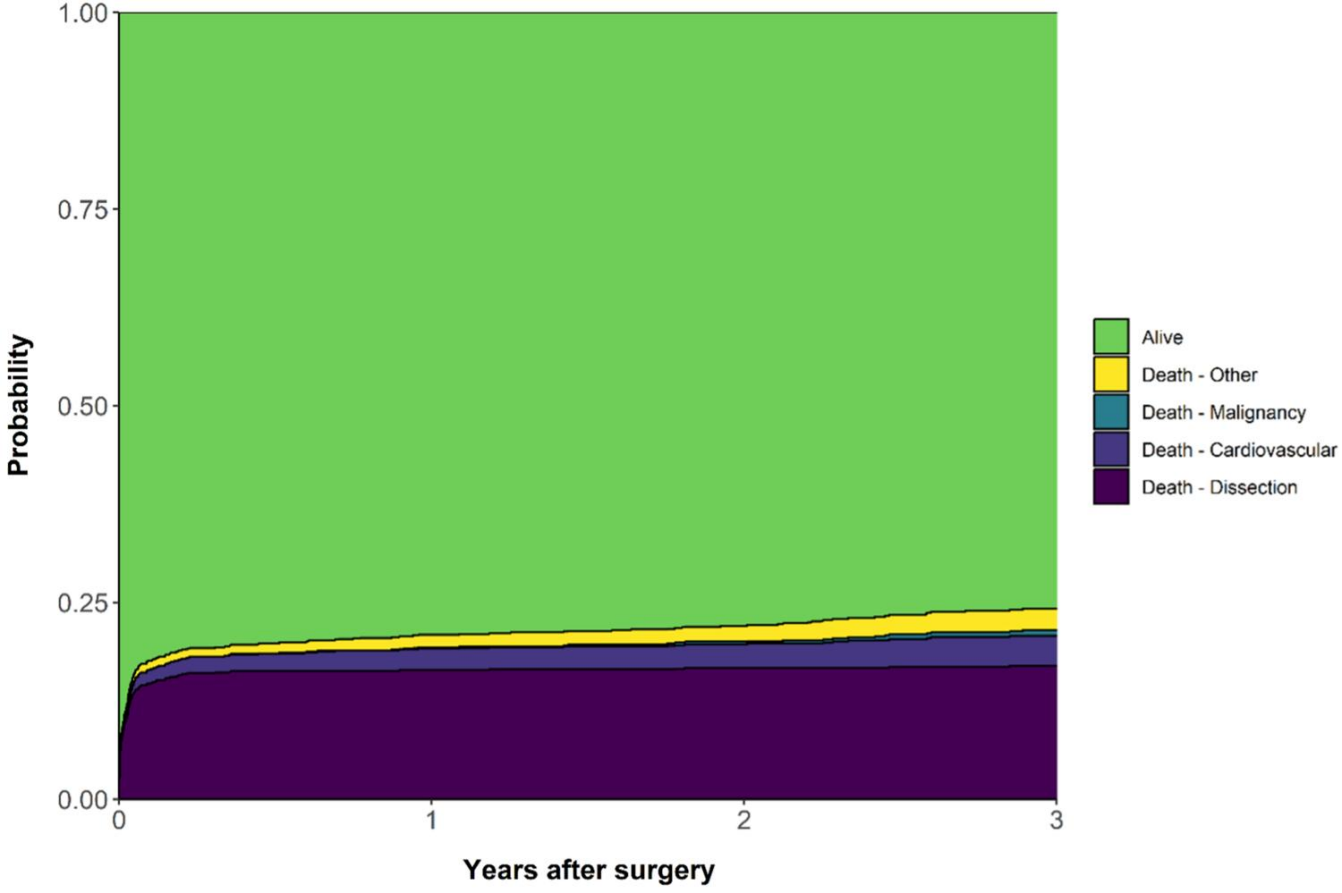
Graphical overview of the causes of death during 3-year follow-up after surgery for type A aortic dissection in the Netherlands. The vertical axis represents the probability to be in one of the defined states. All patients are in the ‘Alive’ state at the start of follow-up and move to one of the death states when they die of that particular cause.

A total of 318 patients died during follow-up and of them 226 (71.1%) died within 30 days. An overview of all the causes of death is provided throughout the entire study as well as during specific time periods in Table 3. Most of the patients who died, died from the consequences of their dissection (*N* = 222, 69.8%). Interestingly, whereas 85% of the deaths within 30 days were attributed to dissections, only 14% of the deaths after 365 days were. Specifically, 365-day survivors died from other cardiovascular disease in 37%, malignancy in 16% and other undefined causes in 33%. Figure 3 illustrates this relation between the cause of death and survival time. The location of death is presented in Table 4 similar to the cause of death. In total, 267 (84%) patients died in the hospital, and after 365 days, a considerable amount of the deaths occurred at home or in a nursing home.

**Table 3:** Causes of death for patients who died after surgery for acute type A aortic dissection in the Netherlands between 2018 and 2021

Cause of death	Overall; *N* = 318	<30 days; *N* = 226	30–365 days; *N* = 49	>365 days; *N* = 43
Dissection	222 (70%)	190 (84%)	26 (53%)	6 (14%)
Cardiovascular	52 (16%)	22 (9.7%)	14 (29%)	16 (37%)
Malignancy	9 (2.8%)	14 (6.2%)^a^	9 (18.1%)^a^	7 (16%)
Other	35 (11%)			14 (33%)

Data are presented as *n* (%).

a To protect privacy, low number of table entrances are combined.

**Table 4:** Location of death for patients who died after surgery for acute type A aortic dissection in the Netherlands between 2018 and 2021

Location of death	Overall; *N* = 318	<30 days; *N* = 226	30–365 days; *N* = 49	>365 days; *N* = 43
Hospital	267 (84%)	220 (97%)	32 (65%)	15 (35%)
Home	26 (8.2%)	6 (3)^a^	9 (18%)	16 (37%)
Nursing home	12 (3.8%)		4 (8.2%)	8 (19%)
Other	7 (2.2%)		4 (8.1%)^a^	4 (9.3%)^a^
Unknown	6 (1.9%)			

Data are presented as *n* (%).

a To protect privacy, low number of table entrances are combined.

## DISCUSSION

This nationwide registry study demonstrates that in-hospital mortality after surgery for type A dissection in The Netherlands has decreased over recent years, while the number of surgeries has increased. Furthermore, patients who survive 1 year after surgery seldom die from the consequences of their dissection but die from other causes, such as cardiovascular diseases and cancer. This could be important information for the surgical and follow-up strategy.

In our study, the 30-day mortality rate was 17.2% over the years 2018–2021. A recent nationwide Danish study reported a 30-day mortality rate of 24.0% between 2011 and 2016 [14]. The IRAD reported an overall in-hospital mortality of 18.4% for surgically treated TAAD patients between 2010 and 2013 [7], the Scandinavian multicentre study NORCAAD of 15.7% for 2005–2014 [11] and the nationwide German study GERAADA of 20.2% for 2006–2010 [17]. These mortality rates are difficult to compare as the corresponding surgical intervention year and study setting differ.

Our nationwide data show a steep decrease in in-hospital mortality from 20.4% in 2018 to 13.9% in 2021, which persisted after adjustment for age and sex. Such a significant improvement in short-term survival in only 4 years is hard to explain from a clinical standpoint. Possibly, although the number of surgeries increased, the selection of patients may have contributed to this finding. Moreover, the COVID-19 pandemic might also have influenced the results. The dissection registry of the NHR is currently expanding with more data including detailed periprocedural characteristics, which will help better understand the observed improvements in outcomes in the future.

In a nationwide Danish study, the recorded deaths within 5 years of the initial TAAD operation were stratified into early death (≤30 days) and non-early death (>30 days) [14]. For the early deaths, 57%, 1.7% and 4.1% were aortic-, cardiac- and cancer-related, respectively. For non-early deaths, the authors reported 23.2%, 3.1% and 12.8% due to aortic-, cardiac- and cancer-related deaths, respectively. These trends are comparable to our results, particularly the significant rise in cancer- and cardiac-related deaths and reduction in dissection-related deaths.

Recently, aortic root replacement (ARR) has been described as a more extensive approach in TAAD operations that should be considered, even in an acute setting, in patients with acknowledged risk factors for late aortic complications or probable need for reinterventions [20]. Moreover, ARR is recommended by current guidelines to be performed in patients where dissection involves the root, and operative risk is acceptable [3]. In the Netherlands, ARR was performed on 32.5% of all operations. ARR was reported in 30.8% in IRAD, 28.3% in GERAADA and 26% in NORCAAD [7, 15, 21].

The standard approach is an open distal anastomosis in most cases with a hemiarch replacement. Conflicting results have been published, but some studies have argued that complete arch replacement leads to decreased mortality and fewer late reinterventions [4, 17, 22–24]. A meta-analysis by Poon *et al.* [25] demonstrated no difference in mortality for patients who underwent hemiarch versus total arch repair. Partly conflicting, another meta-analysis by Ma *et al.* [4] showed that hemi-arch had lower early mortality rates than total arch repair, but higher late mortality rates. We observed in our study that, on average, in the Netherlands, 10% of all TAAD surgeries included complete arch replacement. The GERAADA study reported a higher, 16.1% complete arch, with 47.5% hemi arch reconstruction [17]. The IRAD study reported an increase in complete arch replacement from 16.4%, 21.5% and 22.1% for 1996–2003, 2004–2009 and 2010–2016, respectively [7, 9]. Surprisingly, the NORDCAAD reported only 6% full arch for 2005–2014 [21]. These results show that the extent of arch is subject to discussion, as recently highlighted in current guidelines [2, 3].

The priority of TAAD operation is short-term survival, but extensive root and arch operations are performed in routine practice. It seems from our results that TAAD surgeries in the Netherlands extensively utilize a root approach while adopting a more conservative approach for the arch. Extensive surgery may be beneficial for patients in the long run, but further research is needed.

### Strengths and limitations

One of the key strengths of this study is the nationwide aspect. This creates a broad overview of the entire nation’s healthcare system for a relatively rare disease, which is only possible in a medical system that has unified electronic health record or reliable nationwide registries. In addition, we observe very low rates of missing data. Yet, the inherent limitation when conducting nationwide registry-based studies is the validity of the registered diagnostic codes, for example in ICD-10 codes and the risk of misclassification/information bias in data of Statistics Netherlands. This information is often completed by general practitioners, and without an autopsy, the true cause of death may be challenging to accurately determine. Finally, a minor limitation is that 2% of all TAAD could not be matched and were therefore excluded.

This study did not include data on all patients diagnosed with TAAD in the Netherlands, but exclusively those patients who underwent surgery. Hence, the results could be influenced by patient selection, especially during the COVID-19 pandemic. However, another nationwide study from the Netherlands reported no difference in overall number of acute aortic operations during the pandemic [26] and we saw an increase in surgeries over the 4-year study period.

## CONCLUSIONS

This nationwide registry study demonstrates that in-hospital mortality after surgery for type A dissection in the Netherlands has decreased in recent years. Patients who survive 1 year after surgery seldom die from the consequences of their dissection but die from other causes, such as cardiovascular diseases and cancer. These new insights are valuable for patients and for clinicians who provide treatment and for follow-up guidelines.

## Supplementary Material

ivaf009_Supplementary_Data

## Data Availability

Data are available upon reasonable request to the corresponding author and representatives of the data sources.

## Notes

Presented at the 38th EACTS Annual Meeting (E-Poster).

## ABBREVIATIONS

ARR: Aortic root replacement
CBS: Centraal Bureau voor de Statistiek
GERAADA: German Registry for Acute Aortic Dissection Type A
ICD-10: International Classification of Disease—10
IRAD: International Registry of Acute Aortic Dissection
NHR: Netherlands Heart Registration
NORCAAD: Nordic Consortium for Acute Type A Aortic Dissection
TAAD: Type A aortic dissection

## References

[1] Carrel T , Sundt TM 3rd, von Kodolitsch Y, Czerny M. Acute aortic dissection. Lancet 2023;401:773–88. 36640801 10.1016/S0140-6736(22)01970-5

[2] Czerny M , Grabenwöger M, Berger T et al; EACTS/STS Scientific Document Group. EACTS/STS Guidelines for diagnosing and treating acute and chronic syndromes of the aortic organ. Eur J Cardiothorac Surg 2024;65:5–115. 10.1093/ejcts/ezad426 38408364

[3] Mazzolai L , Teixido-Tura G, Lanzi S et al; ESC Scientific Document Group. 2024 ESC Guidelines for the management of peripheral arterial and aortic diseases. Eur Heart J 2024;45:3538–700. 39210722 10.1093/eurheartj/ehae179

[4] Ma L , Chai T, Yang X et al Outcomes of hemi- vs. total arch replacement in acute type A aortic dissection: a systematic review and meta-analysis. Front Cardiovasc Med 2022;9:988619. 36237909 10.3389/fcvm.2022.988619 PMC9552831

[5] Pedersen MW , Kragholm K, Oksjoki R et al Characteristics and outcomes in patients with acute aortic dissection: a nationwide registry study. Ann Thorac Surg 2023;116:1177–84. 37419172 10.1016/j.athoracsur.2023.06.019

[6] Pape LA , Awais M, Woznicki EM et al Presentation, diagnosis, and outcomes of acute aortic dissection: 17-year trends from the International Registry of Acute Aortic Dissection. J Am Coll Cardiol 2015;66:350–8. 26205591 10.1016/j.jacc.2015.05.029

[7] Evangelista A , Isselbacher EM, Bossone E et al; IRAD Investigators. Insights from the International Registry of Acute Aortic Dissection: a 20-year experience of collaborative clinical research. Circulation 2018;137:1846–60. 29685932 10.1161/CIRCULATIONAHA.117.031264

[8] Arnaoutakis GJ , Wallen TJ, Desai N et al Outcomes of acute type A aortic dissection during the COVID-19 pandemic: an analysis of the Society of Thoracic Surgeons Database. J Card Surg 2022;37:4545–51. 36378930 10.1111/jocs.17085

[9] Trimarchi S , Nienaber CA, Rampoldi V et al; International Registry of Acute Aortic Dissection Investigators. Contemporary results of surgery in acute type A aortic dissection: the International Registry of Acute Aortic Dissection experience. J Thorac Cardiovasc Surg 2005;129:112–22. 15632832 10.1016/j.jtcvs.2004.09.005

[10] Pupovac SS , Hemli JM, Bavaria JE et al Moderate versus deep hypothermia in type A acute aortic dissection repair: insights from the International Registry of Acute Aortic Dissection. Ann Thorac Surg 2021;112:1893–9. 33515541 10.1016/j.athoracsur.2021.01.027

[11] Geirsson A , Ahlsson A, Franco-Cereceda A et al The Nordic Consortium for Acute type A Aortic Dissection (NORCAAD): objectives and design. Scand Cardiovasc J 2016;50:334–40. 27615395 10.1080/14017431.2016.1235284

[12] Chemtob RA , Fuglsang S, Geirsson A et al Stroke in acute type A aortic dissection: the Nordic Consortium for Acute Type A Aortic Dissection (NORCAAD). Eur J Cardiothorac Surg 2020;58:1027–34. 32688394 10.1093/ejcts/ezaa197

[13] Howard DP , Banerjee A, Fairhead JF, Perkins J, Silver LE, Rothwell PM; Oxford Vascular Study. Population-based study of incidence and outcome of acute aortic dissection and premorbid risk factor control: 10-year results from the Oxford Vascular Study. Circulation 2013;127:2031–7. 23599348 10.1161/CIRCULATIONAHA.112.000483 PMC6016737

[14] Obel LM , Lindholt JS, Lasota AN et al Clinical characteristics, incidences, and mortality rates for type A and B aortic dissections: a nationwide Danish population-based cohort study from 1996 to 2016. Circulation 2022;146:1903–17. 36321467 10.1161/CIRCULATIONAHA.122.061065

[15] Kallenbach K , Büsch C, Rylski B et al Treatment of the aortic root in acute aortic dissection type A: insights from the German Registry for Acute Aortic Dissection Type A (GERAADA) registry. Eur J Cardiothorac Surg 2022;ezac261. 10.1093/ejcts/ezac261 35441677

[16] Kurz SD , Falk V, Kempfert J et al Insight into the incidence of acute aortic dissection in the German region of Berlin and Brandenburg. Int J Cardiol 2017;241:326–9. 28499667 10.1016/j.ijcard.2017.05.024

[17] Easo J , Weigang E, Hölzl PP et al Influence of operative strategy for the aortic arch in DeBakey type I aortic dissection—analysis of the German Registry for Acute Aortic Dissection type A (GERAADA). Ann Cardiothorac Surg 2013;2:175–80. 23977579 10.3978/j.issn.2225-319X.2013.01.03 PMC3741835

[18] Timmermans MJC , Houterman S, Daeter ED et al; PCI Registration Committee of the Netherlands Heart Registration and the Cardiothoracic Surgery Registration Committee of the Netherlands Heart Registration. Using real-world data to monitor and improve quality of care in coronary artery disease: results from the Netherlands Heart Registration. Neth Heart J 2022;30:546–56. 35389133 10.1007/s12471-022-01672-0 PMC8988537

[19] Houterman S , van Dullemen A, Versteegh M et al; PCI Registration Committee of the Netherlands Heart Registration. Data quality and auditing within the Netherlands Heart Registration: using the PCI registry as an example. Neth Heart J 2023;31:334–9. 36645544 10.1007/s12471-022-01752-1 PMC10444924

[20] Arabkhani B , Verhoef J, Tomšič A, van Brakel TJ, Hjortnaes J, Klautz RJM. The aortic root in acute type A dissection: repair or replace? Ann Thorac Surg 2023;115:1396–402. 35870520 10.1016/j.athoracsur.2022.06.041

[21] Geirsson A , Shioda K, Olsson C et al Differential outcomes of open and clamp-on distal anastomosis techniques in acute type A aortic dissection. J Thorac Cardiovasc Surg 2019;157:1750–8. 30401530 10.1016/j.jtcvs.2018.09.020

[22] Yang B , Norton EL, Shih T et al Late outcomes of strategic arch resection in acute type A aortic dissection. J Thorac Cardiovasc Surg 2019;157:1313–21.e2. 30553592 10.1016/j.jtcvs.2018.10.139 PMC6441394

[23] Biancari F , Lega JR, Mariscalco G et al Aortic arch surgery for DeBakey type 1 aortic dissection in patients aged 60 years or younger. BJS Open 2024;8:zrae047. 10.1093/bjsopen/zrae047 PMC11104530 38768283

[24] Permanyer E , Ruyra X, Evangelista A. The aortic arch management for type A aortic dissection: aggressive but experienced. J Thorac Dis 2020;12:3429–32. 32642271 10.21037/jtd.2020.01.58 PMC7330748

[25] Poon SS , Theologou T, Harrington D, Kuduvalli M, Oo A, Field M. Hemiarch versus total aortic arch replacement in acute type A dissection: a systematic review and meta-analysis. Ann Cardiothorac Surg 2016;5:156–73. 27386403 10.21037/acs.2016.05.06 PMC4893527

[26] de Beaufort HWL , Roefs MM, Daeter EJ, Heijmen RH; Cardiothoracic Surgery Registration Committee of the Netherlands Heart Registration. Impact of the coronavirus disease 2019 pandemic on volume of thoracic aortic surgery on a national level. Eur J Cardiothorac Surg 2022;61:854–9. 34986237 10.1093/ejcts/ezab550 PMC8755400

