# Corrigendum to: Survival outcomes after surgery for type A aortic dissection: a contemporary Dutch nationwide registry study

**DOI:** 10.1093/icvts/ivaf137

**Published:** 2025-06-16

This is a Corrigendum to: Patrick T Timmermans, Bart J J Velders, Rolf H H Groenwold, Roemer J Vos, Maaike M Roefs, Jerry Braun, Robert J M Klautz, Gianclaudio Mecozzi, Jesper Hjortnaes, Cardiothoracic Surgery Registration Committee and the Aortic Surgery Work Group of the Netherlands Heart Registration, Survival outcomes after surgery for type A aortic dissection: a contemporary Dutch nationwide registry study, *Interdisciplinary CardioVascular and Thoracic Surgery*, Volume 40, Issue 3, March 2025, https://doi.org/10.1093/icvts/ivaf009

The following changes have been made to the originally published manuscript. The **ACKNOWLEDGEMENTS** section of the article is emended to read: […] the Aortic Surgery Work Group of the Netherlands Heart Registration (Wilson Li, Guillaume Geuzebroek, Ron Speekenbrink, Sander Bramer, Sandeep Singh, Khalil Ayan, Egidius van Aarnhem, Edgar Daeter, Elham Bidar, Jos Bekkers, Thomas van Brakel, Aanke Bijleveld, Aria Yazdanbakhsh, Antoine Driessen) for their efforts to develop a nationwide registry and to collect the data.” instead of: “[…]the Aortic Surgery Work Group of the Netherlands Heart Registration for their efforts to develop a nationwide registry and to collect the data.”

