# Supplementary material for: Survival outcomes after surgery for type A aortic dissection: a contemporary Dutch nationwide registry study
Source: Interdiscip Cardiovasc Thorac Surg. 2025 Feb 6;40(3):ivaf009. doi: 10.1093/icvts/ivaf009 (PMC11893151; doi:10.1093/icvts/ivaf009)
Supplement: ivaf009_Supplementary_Data [file ivaf009_supplementary_data.docx]

**Data supplementary****: survival outcomes after surgery for type-a aortic dissection: a contemporary Dutch nationwide registry study**

**Supplementary Table 1: Cox regression**

| **Variable** | **HR***^1^* | **95% CI***^1^* | **p-value** |
| --- | --- | --- | --- |
| Intervention year |  |  |  |
| 2018 | — | — |  |
| 2019 | 0.98 | 0.71 1.36 | 0.917 |
| 2020 | 0.92 | 0.65, 1.30 | 0.653 |
| 2021 | 0.89 | 0.63, 1.25 | 0.510 |
| Age | 1.02 | 1.01,1.03 | <0.001 |
| Sex |  |  |  |
| Male | — | — |  |
| Female | 1.09 | 0.87, 1.36 | 0.5 |
| Euroscore II | 1.02 | 1.01, 1.03 | <0.001 |
| *^1^* HR = Hazard Ratio, CI = Confidence Interval | | | |
|  | | | |

**Supplementary Table 2: Logistic regression**

| **Variable** | **OR** | **CI** | **p-value** |
| --- | --- | --- | --- |
| Intervention year |  |  |  |
| 2018 | - | - |  |
| 2019 | 0.88 | 0.56, 1.37 | 0.570 |
| 2020 | 0.88 | 0.56, 1.39 | 0.591 |
| 2021 | 0.68 | 0.43, 1.06 | 0.091 |
| Age | 1.02 | 1.01,1.03 | 0.008 |
| Sex |  |  |  |
| Male | - |  |  |
| Female | 0.99 |  | 0.931 |
| Euroscore II | 1.03 | 1.02, 1.04 | <0.001 |
| *^1^* OR = Odds Ratio, CI = Confidence Interval | | | |

**Supplementary Table 3: Table 1 per operation year**

| **Characteristics** | 2018 | 2019 | | 2020 | 2021 | p-value |
| --- | --- | --- | --- | --- | --- | --- |
| n | 284 | 332 | | 328 | 373 |  |
| Age (years) | 63.02 (11.81) | 63.36 (12.39) | | 62.99 (11.46) | 63.18 (11.59) | 0.979 |
| Male Seks | 115 ( 40.5) | 138 ( 41.6) | | 144 ( 43.9) | 150 ( 40.2) | 0.765 |
| BMI (kg/m^2^) | 26.32 (4.35) | 26.51 (5.35) | | 26.67 (4.40) | 26.44 (4.74) | 0.830 |
| BSA (m^2^) | 1.99 (0.22) | 1.98 (0.24) | | 1.98 (0.24) | 1.98 (0.24) | 0.975 |
| Euroscore II | 11.10 [6.47, 20.71] | 10.53 [5.42, 20.83] | | 9.17 [5.30, 17.12] | 9.72 [5.08, 19.72] | 0.057 |
| LVEF (%) | 53.61 (6.18) | 53.80 (6.82) | | 54.25 (5.38) | 52.86 (6.78) | 0.038 |
| Creatinine (umol/l) | 100.60 (44.61) | 96.39 (40.11) | | 96.71 (48.38) | 98.69 (53.09) | 0.682 |
| Diabetes mellitus | 8 ( 3.0) | 11 ( 3.3) | | 15 ( 4.3) | * | 0.033 |
| Atrial fibrillation | 31 ( 12.4) | 18 ( 7.0) | | 18 ( 6.5) | 5 ( 10.4) | 0.038 |
| Connective tissue disease | 15 ( 7.2) | 18 ( 7.9) | | 16 ( 6.7) | 15 ( 5.5) | 0.729 |
| Chronic lung disease | 11 ( 3.9) | 22 ( 6.6) | | 24 ( 7.4) | 32 ( 8.6) | 0.112 |
| Previous CVA | 11 ( 3.9) | 15 ( 4.5) | | 13 ( 4.1) | 23 ( 6.3) | 0.427 |
| Previous ECVD | 50 ( 17.6) | 61 ( 18.4) | | 51 ( 15.7) | 68 ( 18.3) | 0.775 |
| Previous cardiac surgery | 16 ( 5.6) | 19 ( 5.7) | | 11 ( 3.4) | 23 ( 6.2) | 0.358 |
| Critical state | 98 ( 34.5) | 99 ( 29.9) | | 91 ( 27.8) | 104 ( 28.0) | 0.239 |
| Urgency setting |  |  | |  |  | <0.001 |
| Urgent | 23 ( 8.1) | 34 ( 10.2) | | 31 ( 9.5) | 37 ( 9.9) |  |
| Emergency | 203 ( 71.5) | 256 ( 77.1) | | 260 ( 79.3) | 297 ( 79.6) |  |
| Salvage | 58 ( 20.4) | 42 ( 12.7) | | 37 ( 11.3) | 39 ( 10.5) |  |
| n (%); mean (±sd); median [interquartile range] | | |  |  |  |  |
| Abbreviations: BMI = Body Mass Index, BSA = Body Surface Area, CVA = Cerebrovascular Accident, ECVD = Extracardiac Vascular Disease, DOAC = Direct Oral Anticoagulant, LVEF = Left Ventricular Ejection Fraction | | | | | | |

**Supplementary Table 4: Table 2 per operation year**

| **Characteristics** | 2018 | 2019 | 2020 | 2021 | p-value |
| --- | --- | --- | --- | --- | --- |
| n | 284 | 332 | 328 | 373 |  |
| CPB time (min) | 265.15 (101.19) | 257.94 (103.83) | 251.51 (102.97) | 256.31 (98.73) | 0.462 |
| Cross clamp time (min) | 147.07 (66.29) | 141.41 (69.71) | 133.12 (68.14) | 144.07 (71.81) | 0.088 |
| Circulatory arrest | 253 ( 89.1) | 277 ( 83.4) | 287 ( 88.0) | 344 ( 92.2) | 0.004 |
| Circulatory arrest time (min) | 44.70 (30.47) | 44.47 (29.89) | 42.23 (27.85) | 43.37 (27.14) | 0.732 |
| Concomitant CABG | 16 ( 5.6) | 29 ( 8.7) | 26 ( 7.9) | 36 ( 9.7) | 0.295 |
| TAAD surgery extend |  |  |  |  |  |
| AV repair or replacement | 109 ( 38.4) | 129 ( 38.9) | 129 ( 39.3) | 151 ( 40.5) | 0.951 |
| Root | 93 ( 33.0) | 103 ( 31.0) | 102 ( 31.1) | 130 ( 34.9) | 0.965 |
| Aortic Arch | 214 ( 75.4) | 230 ( 69.3) | 243 ( 74.1) | 274 ( 73.5) | 0.339 |
| Descending aorta | * | * | * | * | * |
| Minimum central temperature | 23.13 (4.11) | 23.57 (4.39) | 23.57 (4.06) | 23.49 (3.52) | 0.524 |
| Number of arch vessels repaired |  |  |  |  | 0.010 |
| 0 vessels | 201 ( 71.5) | 232 ( 73.4) | 240 ( 76.4) | 280 ( 76.1) |  |
| 1 vessel | 12 ( 4.3) | 13 ( 4.1) | 13 ( 4.1) | 5 ( 1.4) |  |
| 2 vessels | 26 ( 9.3) | 37 ( 11.7) | 38 ( 12.1) | 54 ( 14.7) |  |
| 3 vessels | 42 ( 14.9) | 34 ( 10.8) | 23 ( 7.3) | 29 ( 7.9) |  |
| n (%); mean (± sd). Times are reported in minutes.  Abbreviations: AV = Aortic Valve, CABG = Coronary Artery Bypass Graft, CPB: Cardiopulmonary bypass | | | | | |
| *To protect privacy, low number of table entrances are combined. | | | |  |  |
